# The Impact of China’s Legal System on Public Health and Quality of Life during the COVID-19 Pandemic: An Empirical Study

**DOI:** 10.3390/ijerph192013598

**Published:** 2022-10-20

**Authors:** Weiwei Duan, Tianbao Qin

**Affiliations:** 1 School of Humanities and Law, Zhengzhou University of Aeronautics, Zhengzhou 450046, China; 2 Research Institute of Environmental Law, Wuhan University, Wuhan 430072, China

**Keywords:** COVID-19, legal system, public health and quality of life, China

## Abstract

(1) Background: During the COVID-19 pandemic, the methods used to ensure the quality of public health and life changed. This study used empirical research methods to analyze the development of China’s legal system during the COVID-19 pandemic and its impact on the quality of public health and life and to clarify its importance in ensuring the quality of public health and life. (2) Materials and Methods: This study analyzed the development of China’s rule of law system during the COVID-19 pandemic by using two authoritative databases, the Peking University Magic Key Database and the China Judicial Documents Network, and examined the scope of public health and quality of life under this system. (3) Results: During the COVID-19 pandemic, China’s rule of law system, with the main objective of controlling the pandemic, developed rapidly, effectively ensuring the quality of public health and life in different areas. As part of this system, the administrative activities of government departments played a key role. (4) Conclusions: During the period of the COVID-19 pandemic, China’s legal system protected the quality of public health and life from the impact of the pandemic in many areas. Meanwhile, along with the important role of government administrative law enforcement activities in the prevention and control of a pandemic, the quality of public health and life should also be protected against illegal administrative acts in the future.

## 1. Introduction

Public health and quality of life mainly refer to the state of physical and mental health that citizens or organizations can perceive over time [1]. Presently, the main research fields of public health and quality of life include physical health, mental health, social function, role function, and overall health [2]. Since the COVID-19 pandemic was confirmed in 2020 [3], China gained valuable experience in coping with the virus and the ensuing pandemic [4]. During the period of the COVID-19 pandemic, more factors have affected the quality of public health and life. In addition to meeting the basic physical health need of citizens and mitigating the impact of the pandemic [5], government departments should also protect the people’s mental health [6], personal quality of life [7], and other related areas. As such, it is necessary to take various emergency management measures to protect the quality of public health and life from the impact of the pandemic [8].

In response to the emergency generated by the pandemic, active legal interventions not only provide beneficial support for controlling the pandemic [9] but can bring the quality of public health and life under the protection of the rule of law so as to achieve coordination between the government’s pandemic emergency management activities and its responsibility to meet public health and quality needs. In the context of the multifaceted impact of the COVID-19 pandemic on global development [10], China has attached great importance to maximizing the government’s public management capacity to cope with the adverse effects [11]. Additionally, the country has made beneficial contributions to the protection of global public health and quality of life [12].

During the COVID-19 pandemic, the Chinese leadership and government adopted scientific prevention and control measures that allowed China to achieve remarkable results in preventing and controlling the spread of SARS-CoV-2 [13]. In particular, the continuous improvement of the legal system has significantly guaranteed the quality of public health and life in many aspects. China’s effective pandemic prevention and control actions have won the trust and support of its citizens, and the public trust in the government is far higher than the average global level [14]. However, it should still be noted that government departments have gained more discretion based on the need for pandemic prevention and control [15], and public health and quality of life are vulnerable to the impact of illegal administrative acts by the government [16]. In this context, preventing the governmental from acting illegally and government departments from neglecting the protection of public health and quality of life is also the focus of China’s legal system for the future.

This study aimed to explore how the rule of law system in China’s pandemic prevention and control work affects the public health and quality of life. It mainly discusses how China’s legal system guarantees the public health and quality of life through legislation, law enforcement and justice during the pandemic, and which aspects are affected by the system. This study mainly used an empirical research method to discuss the relationship between the legal system and the public health and quality of life in China. In the study, we explained which major public healthy life rights were specified in the legal provisions and discussed how these rights were specifically safeguard by the administrative law enforcement and the judicial trial mechanisms. At the same time, we analyzed the development of the legal system before and after the pandemic to assess the main ways and scope to which legal system ensured the public health and quality of life. The objective of this study is to illustrate to policy makers, relevant stakeholders and the public the important value of legal system in ensuring the public health and quality of life during the pandemic, and appeal to government departments for implementation of pandemic prevention according to the law, so as to avoid adverse effects on the public health and quality of life.

## 2. Materials and Methods

### 2.1. Data Source

To ensure the comprehensiveness and authenticity of the research data, we obtained specific data on the construction of the legal system, such as laws and regulations, normative documents, working documents, and administrative law enforcement cases, during the COVID-19 pandemic in China through the Peking University Magic Key Database. We obtained criminal cases, civil cases, and administrative cases related to the pandemic tried by Chinese judicial institutions during the pandemic through the China Judicial Documents Network, in order to understand the specific situation of the legal system regarding the protection of public health and quality of life during the pandemic.

### 2.2. Research Sample

During the COVID-19 pandemic in China, changes in the legal system for pandemic prevention and control had an impact on the quality of public health and life. The rule of law had new changes focusing on ways to ensure the quality of public health and life, leading to changes in their relationship. This study focused on the real relationship between China’s legal system and the quality of public health and life during the COVID-19 pandemic. The system composition and practical effect of China’s legal system were studied. The research samples included legal systems, normative documents, working documents, administrative law enforcement cases, and judicial cases related to pandemic prevention and control before and after the SARS-CoV-2 outbreak in China. In this context, quality of public health and life mainly refers to the specific situation where it is guaranteed by the rule of law in different areas, such as medicine and health, food and drugs, public safety, and the living environment, during the pandemic. The study was conducted to obtain the real connection between the Chinese legal system and the quality of public health and life.

### 2.3. Procedure

In this study, we summarize the development data of China’s pandemic prevention and control legal system and compare its overall development before and after the COVID-19 outbreak in terms of the legislative system, law enforcement mechanism, and judicial trial practice. We analyzed the main areas of the legal system for pandemic prevention and control to ensure the quality of public health and life during the COVID-19 pandemic. We also use specific examples to discuss the methods and value of the legislative system, law enforcement mechanism, and judicial trial practice in terms of ensuring the quality of public health and life, and obtain the specific effect of the pandemic prevention and control legal system on the process.

We selected specific data of China’s legal system, law enforcement activities, and judicial cases related to pandemic prevention and control during the pandemic to analyze the framework of the legal system during the pandemic. We also focused on analyzing the development of and changes to the legal system before and after the SARS-CoV-2 outbreak. This research allowed us to obtain the overall framework of China’s legal system as well as find out its main components and development priorities during the COVID-19 pandemic.

During the pandemic, the quality of public health and life was affected, not only in terms of health, but also in areas such as learning and education [17], daily diet [18], and working environment [19]. This study analyzed the protection scope of legal means for ensuring the quality of public health and life, including medical and health, food and drugs, public safety, and other related areas. This not only clarifies the importance of China’s legal system in ensuring the quality of public health and life during the COVID-19 pandemic but also promotes a new understanding of the issue.

Against the background of remarkable results achieved in controlling the COVID-19 pandemic [20], this study focused on the factors in China’s legal system that had a great impact on the quality of public health and life during the pandemic, especially the possible adverse effect of the expansion of law enforcement power of government departments, and analyzed the relationship between the quality of public health and life and the pandemic prevention behavior of government departments.

## 3. Results

### 3.1. China’s Legal System, Normative Documents, and Working Documents Related to Pandemic Prevention and Control

Before the outbreak of SARS-CoV-2, the legislative system stipulated the public’s rights in many aspects related to quality of life, and also clarified the government’s right to ensure the public’s health. For example, Article 21 of the Constitution of the People’s Republic of China stipulates that the state has the responsibility to develop medical and health services and protect people’s health. Articles 1002, 1003, and 1004 of the Civil Code of the People’s Republic of China stipulate that citizens may enjoy the right to life, health, and body and are protected by law. The Law of the People’s Republic of China on the Prevention and Control of Infectious Diseases clarifies in Article 1 the legislative purpose of the law to protect human health and public health, and also clarifies the responsibilities of the government in terms of infectious disease prevention, pandemic prevention, pandemic notification, pandemic control, medical treatment, etc. At the same time, the Emergency Response Law of the People’s Republic of China, the Emergency Response Regulations for Public Health Emergencies, and other laws also provide a legal basis for government departments to deal with the pandemic while protecting the public’s right to life, health, body, and information.

After the outbreak of the new coronavirus, the National Health Commission included COVID pneumonia in the Class B infectious diseases specified in the Law on the Prevention and Control of Infectious Diseases and took prevention and control measures against Class A infectious diseases, making the Law on the Prevention and Control of Infectious Diseases an important legal basis for China’s pandemic prevention and control efforts. The Biosafety Law, which was implemented in April 2021, also clearly defines the main purpose of protecting people’s lives and health, and it stipulates the means and legal responsibilities for preventing and controlling new major infectious diseases. In addition, during the pandemic, in order to further uphold rights related to quality of life, such as the right to life, to health, and to the body, which are guaranteed by the legislative system, China also issued many normative and working documents, which are more flexible in their formulation and more targeted in content than laws. Although these documents do not fall within the scope of the legal system in form, they play an important role in implementing legal provisions and guiding government departments to carry out pandemic prevention and control.

This study analyzed the laws, administrative regulations, judicial interpretations, departmental rules, local regulations, local government rules, and other legal systems related to pandemic prevention and control in China, before and after the SARS-CoV-2 outbreak, using the Peking University Magic Key Database. The data showed that before the outbreak, China had a relatively complete legal system for pandemic prevention and control. The legal norms in the system were mainly promulgated by the National People’s Congress and its Standing Committee, the State Council, the Supreme People’s Court, and the Supreme People’s Procuratorate, departments under the State Council, local people’s congresses and standing committees, and local people’s governments. By July 2022, more than two years after the outbreak began, the overall number of laws aimed at pandemic prevention and control had significantly increased, with local legislation and government regulations issued by local institutions with legislative power accounting for most of them (see Table 1).

Normative documents are often issued by authorized subjects according to the law; these documents involve public rights and obligations, have universal binding force, and are repeatedly applied within a certain period. During the COVID-19 pandemic, the number of normative documents was far more than that typically found in the legal system. The main bodies of these documents generally included the State Council, the departments under the State Council, and local institutions with legislative power. At the same time, since the normative documents are more flexible than the legal system in terms of the specific content, scope of action, and formulation procedures, and because they have universal binding force, their number increased significantly during the pandemic, which played an important supplementary role in the legal system (see Table 2).

During the COVID-19 pandemic, in addition to the legal system and normative documents, working documents issued by Chinese official departments were also closely related to pandemic prevention and control. Working documents often refer to documents issued by official departments that have no mandatory binding force, such as organizational management and operation, external information release, and activity organization. Such documents are more flexible than normative documents in terms of formulation procedures and specific content and they pay attention to the construction work of the issuing body, which can also reflect the work focus of the issuing body within a certain period. During the pandemic period, the number of working documents issued by Chinese official departments was far more than before the pandemic, and most of them were concentrated in relevant local legislative bodies and administrative departments (see Table 3).

After the outbreak, the number of legislation system for pan-demic prevention and control in China increased significantly, which also reflects the growing influence of laws on major areas involving the quality of public health and life. In this process, the law clarifies not only the right of the public to enjoy a healthy life in different areas, but also the responsibilities of government departments in pandemic prevention and control in different areas, so as to reduce the impact of a pandemic or other events on the quality of public health and life as much as possible.

During the COVID-19 pandemic, the public’s demand for quality of life, in addition to basic physical health, included factors such as a safe social and living environment, which greatly expanded the public coverage range. According to the laws and relevant documents obtained from China’s legal database, this study found that during the COVID-19 pandemic, at least 30 areas closely related to the quality of public health and life were covered by the legal system and documents related to pandemic prevention and control, which reflects the close relationship between the legal system and the quality of public health and life. The legal system and documents not only covered the health field directly related to the quality of public health and life, but also included production and living order, a stable social security environment, personal development opportunities, and other related aspects (see Table 4).

### 3.2. Administrative Law Enforcement by Government Departments during the COVID-19 Pandemic

During the COVID-19 pandemic, there were many illegal violations of pandemic prevention and control laws, including refusing to cooperate with the registration of personal health information, raising the price of pandemic prevention supplies and living materials, manufacturing and selling counterfeit pandemic prevention supplies, and fabricating and spreading false rumors about the pandemic. Such violations not only damage the right to life, health, and body and other rights related to quality of life, but also have a negative impact on the normal order of social life. In order to ensure the public’s health and quality of life through public health and medical services, daily life tasks, a secure public environment, and other aspects during the pandemic, government departments, in accordance with the Public Security Administration Law, the Law on the Prevention and Control of Infectious Diseases, the Administrative Punishment Law, and other laws and regulations, carried out administrative law enforcement activities against illegal acts that disrupted the order of pandemic prevention and control and the quality of public health and life. Law enforcement actions such as warnings, fines, and public security detention have been used to punish illegal acts involving multiple areas of public health and quality of life. At the same time, these activities adhere to the principle of proportionality of administrative law, so that they simultaneously take into account pandemic prevention and control objectives and protect public rights and interests. For example, the temporary containment measures taken by government departments to control the pandemic put more emphasis on scientific determination of the time and scope; avoiding disclosure of personal information when collecting data for pandemic prevention and control; and providing compensation to rights holders when the property of temporarily requisitioned units and individuals was used for pandemic prevention and control.

During the COVID-19 pandemic, China’s strong and integrated governance model was a key component [21]. As part of this model, administrative penalties are imposed for illegal acts that damage the public order and the legitimate rights and interests of the public according to the law, which was an effective guarantee for the government’s implementation of pandemic prevention and control measures. Law enforcement involves action taken by government departments at all levels against specific illegal acts. It has the characteristics of directness and flexibility, and it can effectively avoid or reduce the impact of various illegal acts on the quality of public health and life. Taking the pandemic situation as the key search term, a search in the Magic Key Database of Peking University found that the law enforcement departments have been involved in many areas of public health and quality of life. During the COVID-19 pandemic, the law enforcement activities of Chinese government departments at all levels increased significantly compared with the period before the outbreak in 2020, especially in areas related to public health and quality of life, such as pandemic prevention and control, public security, market supervision, food, and medicine and medical care (see Table 5).

### 3.3. Judicial Trials in China during the COVID-19 Pandemic Period

During the COVID-19 pandemic, criminal acts that seriously violated pandemic prevention and control provisions were subject to criminal punishment. For example, acts causing the spread of coronavirus or posing a serious risk of spread constitute the crime of hindering the prevention and control of infectious diseases, as stipulated in Article 330 of the Criminal Law of the People’s Republic of China; obstructing state functionaries from carrying out pandemic investigation according to the law by means of violence or threat constitutes the crime of obstructing official duties, as stipulated in Article 277; driving up prices, seeking exorbitant profits, seriously disrupting the market order, and obtaining large amounts of illegal income constitute the crime of illegal business operations, as stipulated in Article 225; and fabricating false pandemic information and spreading it on the Internet and other public places, which seriously disrupts the social order, constitutes the crime of fabricating and intentionally spreading false information, as stipulated in Article 291. These crimes not only seriously disturbed the social order during the pandemic, but also seriously violated the right to life and health and other rights. Chinese courts have tried criminal cases involving pandemic prevention and control in accordance with the law, punished criminal acts that harmed the public interest and the quality of health and life, and demonstrated the important role of judicial bodies in ensuring the quality of public health and life.

According to data from the China Judgment Document Network, after the SARS-CoV-2 outbreak, the number of criminal cases related to pandemic prevention and control increased significantly, which involved not only the protection of personal rights, democratic rights, property rights, and other personal rights directly related to public health and quality of life, but also public safety, market economic order, social management order, and other external environmental aspects that had an indirect effect on the quality of public health and life (see Table 6).

The Civil Code of the People’s Republic of China stipulates that citizens may enjoy not only the right to life, health, and body, but also real rights, creditor’s rights, intellectual property rights, inheritance rights, and other rights. Most of these are closely related to quality of life. During the COVID-19 pandemic, civil activities based on pandemic prevention and control not only covered most areas of social and economic development, but also generated a significant increase in the number of civil disputes. In this context, trials of civil cases involving pandemic prevention and control in accordance with the law reflect not only the value of the judicial trial mechanism in maintaining social and economic order, but also its important role in ensuring public health and quality of life by protecting the legitimate rights and interests of civil subjects at different socioeconomic levels.

Before the pandemic, civil disputes accounted for the majority of judicial cases. During the pandemic, the number of various types of cases increased sharply, reflecting the important role of Chinese judicial bodies in resolving disputes between civil subjects and safeguarding the legitimate rights of the public. During the pandemic, the public’s demand for health and quality of life continued to expand, which caused new disputes. Among them, in addition to contract, labor, infringement, and other types of pre-existing disputes, the people’s courts also tried many dispute cases in the areas of marriage and family [22] and company management [23] to protect the public’s civil rights (see Table 7).

During the COVID-19 pandemic, in order to control the situation and ensure public order, Chinese government departments at all levels gained more administrative law enforcement authority. On the one hand, this was conducive to taking more flexible measures to control the pandemic situation. On the other hand, it also had potential adverse effects on the quality of public health and life. For example, government departments violated the provisions of the Law on the Prevention and Control of Infectious Diseases to protect the public’s right to know by concealing false reporting of pandemic information; violated the provisions of the Administrative Punishment Law on the legal procedures of administrative punishment and arbitrary exercise of the power of administrative punishment; violated laws and regulations on excessive containment, isolation, and other pandemic prevention and control measures, damaging the public’s right to life, health, personal freedom and other rights. In order to prevent the expanding administrative law enforcement power of government departments at all levels to impact the quality of public health and life during the pandemic, the public had the right to bring lawsuits against the administrative organs that illegally exercised their functions and powers in accordance with the Administrative Procedure Law of the People’s Republic of China to protect their legitimate rights and interests. Trials of administrative cases in accordance with the law reflect the important role of China’s judicial adjudication mechanism in supervising administrative organs to exercise their functions and powers in accordance with the law.

During the COVID-19 pandemic, to prevent illegal administrative acts of the government from adversely affecting the legitimate rights of the public, the judicial mechanism reviewed and ruled the administrative acts of the government according to the law. With the expanded scope of administrative activities of government departments at all levels during the COVID-19 pandemic, the public and government departments had divergent views in many areas related to public health and quality of life, including labor and social security, housing demolition, township government administration, and others, while there were fewer cases in culture, sports, food and medicine, education, and other areas (see Table 8).

## 4. Discussion

China’s legal system for pandemic prevention and control clarifies the public’s main rights related to the quality of health and life, such as the right to life, health, and body, the right to work, and the right to property, through legal norms so that these rights are strictly protected by law. The system also encourages government departments at all levels to actively perform their duties by clarifying the legal basis and responsibilities for pandemic prevention and control work, in order to reduce the impact of the pandemic on the quality of public health and life. After the SARS-CoV-2 outbreak, government departments at all levels needed a more specific and effective legal basis for pandemic prevention and control and the scope of the right to public health and quality of life during the pandemic needed to be clearly defined in many areas of society, so the number of related laws, normative documents, and working documents supporting the implementation of laws after the outbreak increased significantly. In terms of the administrative law enforcement mechanism, the quality of public health and life is affected by administrative violations in many aspects, such as medical resources, public security environment, living materials, and public transport. Government departments need to use their administrative law enforcement power to punish these violations. After the outbreak, the number of legal cases involving the quality of public health and life increased, which reflects to some extent that China’s administrative law enforcement mechanism maintained high pressure on illegal acts that disrupt public order and damage public health and life. In terms of the trial mechanism, Chinese judicial bodies safeguarded the right of the public to enjoy a healthy life, including by severely punishing all kinds of crimes against the legitimate rights and interests of the public, providing a judicial guarantee for the public to have a healthy life in different social fields, and preventing administrative bodies from illegally exercising their functions and powers. After the pandemic, legal rights related to the public’s health and quality of life were affected by illegal acts in many social areas, and the number of criminal, civil, and administrative cases related to pandemic prevention and control increased significantly. This was a reflection of the judicial trial mechanism providing the public with rights and ways to deal with various illegal acts, thus ensuring a healthy quality of life.

In the process of coping with the COVID-19 pandemic, the Chinese government took various emergency measures to effectively reduce its negative impact and guarantee the quality of public health and life, further enhancing the public’s satisfaction with and trust in the government [24], which was closely related to the development of the legal system during the pandemic [25]. Before the SARS-CoV-2 outbreak, China had an emergency legal system that involved pandemic prevention and control. Government departments also had previous experience in pandemic prevention and control under emergency conditions. China’s legal system had a certain foundation to manage the pandemic [26]. Since the SARS-CoV-2 outbreak in 2020, Chinese leadership has repeatedly stressed the importance of using the concepts and means of the rule of law for pandemic prevention and control [27]. In less than three years, the legislative system, administrative law enforcement activities, and the scale and number of judicial cases related to pandemic prevention and control increased dramatically, reflecting the positive role of China’s legal system in responding to the pandemic. In addition, the legal system not only involved the basic fields of public health and health care, but also specified the factors that affect the quality of public health and life, such as social security, economic order, living environment, and personal development opportunities for citizens. This reflects the close relationship between China’s legal system and the quality of public health and life during the COVID-19 pandemic.

The main purpose of China’s legislative system is to protect people’s lives and health. It not only clarifies the basic rights of the public during the pandemic through legislation, but also provides a legal basis for government departments to carry out complex pandemic prevention and control work. It uses the rule of law to provide norms of conduct for different subjects with a focus on pandemic prevention and control and public rights protection. When these public rights are infringed by the illegal acts of others, the law provides a legal basis for the public to safeguard their rights and for relevant national departments to punish the illegal acts. In terms of legal system construction, even though the legislative authorities have introduced various legal systems since the SARS-CoV-2 outbreak, it has been difficult for them to fully meet the institutional needs of virus prevention and control due to the stability and lag of laws. In this context, the normative documents issued by government departments at all levels provide institutional supplementation and support for pandemic prevention and control. These normative documents have the advantages of a flexible formulation procedure and specific regulatory scope, enabling the government to respond to various aspects of public health needs during the pandemic, and provide a basis for government departments at all levels to carry out emergency measures for pandemic prevention and control. In addition, although the working documents issued by government departments did not have the general binding force of laws and normative documents, their regulatory targets were generally government departments at all levels, and they provided important guidance for carrying out pandemic prevention and control work. Under the legal requirements of pandemic prevention [28], various legal systems, normative documents, and working documents issued by the relevant departments not only focused on public health, pandemic prevention and control, and other areas involved in basic public health, they also comprehensively regulated the public’s living materials, economic order, and individual development to meet the demand for a healthy quality of life during the pandemic.

As important law enforcers and defenders of social public order [29], government departments are authorized by law to expand their scope of authority according to the needs of pandemic prevention and control. In that context, they have the right to exercise specific functions and powers within the scope of the law, which reflect the obvious characteristics of emergency law enforcement. Meanwhile, administrative law enforcement departments have greatly improved the efficiency and intensity of punishment for illegal acts, such as price gouging, production and sales of fake and inferior medical products, and disturbance of the social order caused by the pursuit of illegal interests. To realize the administrative function of stabilizing the social and economic order and to mitigate the impact of the pandemic on public health, the government should ensure that people have a stable and orderly living environment and should use legal means to severely punish illegal acts that disrupt social production and order. At the same time, when government departments take pandemic prevention and control measures, they should adhere to the principle of proportionality in administrative law, define the bottom line for the derogation of basic rights, and try their best to reduce the impact of such measures on citizens’ rights such as health, property, and personal freedom. Therefore, the government’s protection of the quality of public health and life through administrative law enforcement activities is reflected not only in its necessary regulation of social public affairs according to the law based on the need for pandemic prevention and control, but also in the punishment of acts that infringe on the quality of public health and life according to the law, thus realizing the important value of law enforcement actions in this protection. In addition, since government departments were given extensive law enforcement space during the pandemic period, such authorization not only maximized the social and public management value of government departments, but also highlighted the risk of damaging public interests by illegal or excessive exercise of power [30]. To avoid the situation where the government only pays attention to the effect of pandemic control and neglects the demands of public health and quality of life, it is necessary to emphasize the legitimacy of government law enforcement activities in realizing a connection between the two concerns.

During the COVID-19 pandemic, the value of China’s judicial trial mechanism was reflected in ensuring people’s life and health and the social and economic order in the form of punishing various criminal acts, safeguarding citizens’ legitimate civil rights and interests, and supervising the administrative bodies so they would perform their duties according to the law. In this process, the judicial adjudication mechanism not only protected the people’s right to life, health, and body and other major rights related to quality of life from illegal violations, but also provided legal protection for people to carry out normal social and economic activities, thus reducing the impact of the pandemic on quality of life. When the quality of public health and life is affected by illegal acts, in addition to administrative punishment by government departments according to the law, the judicial trial mechanism also provides an important guarantee for safeguarding people’s legitimate rights and interests. Courts at all levels tried various criminal, civil, and administrative cases during the pandemic period covering many areas such as life and health, property protection, healthcare, public security, and economic order, and also brought judicial initiatives into play by taking measures such as issuing typical cases. They considered issues such as the public’s ability to fulfill legal obligations during the pandemic period as being appropriate, reduced the economic and time costs for judicial proceedings, and ensured that all aspects of public health and quality of life were effectively guaranteed by the judicial trial mechanism.

Although China’s legal system guarantees the quality of public health and life in a diversified and comprehensive way, as government departments gain more freedom to enforce the law, there will also be the risk of infringement by illegal administrative acts by some government departments. It was repeatedly stressed that during the pandemic prevention and control period, departments at all levels should firmly adhere to the principles of pandemic prevention according to the law, avoid excessive and one-sided administrative actions so that people’s normal production and living order is not adversely affected, and especially pay attention to protecting people’s legitimate personal freedoms and property rights. The government also paid attention to gaining public understanding of its pandemic prevention and control work by disseminating information [31], which provided ideas for supervising the use of administrative power according to the law through public participation during future pandemics. Government departments at all levels should not illegally expand their restrictions on people’s individual rights based on pandemic prevention and control [32]. At the same time, with the gradual improvement of the rule of law system with pandemic prevention and control as the main objectives, and with the expansion of the administrative law enforcement power of government departments during the pandemic, the quality of public health and life will also face the risk of excessive government administration and illegal administration. In the future, supervising the administration of government departments according to the law and taking reasonable administrative actions for pandemic prevention and control will also be important components of China’s rule of law system during a pandemic.

## 5. Strengths and Limitations

### 5.1. Strengths

First, we obtained the development data and characteristics of China’s legal system during the COVID-19 pandemic through two authoritative legal databases. Second, we classified the Chinese legal system and comprehensively examined its guarantee of public health and quality of life in different areas. Finally, we proposed that China’s legal system should prevent illegal activities by government departments from having an adverse impact on the quality of public health and life in the future and enrich the actual quality of public health and life during a pandemic.

### 5.2. Limitations

First of all, data from before July 2022 were used in this study. Over time, the relevant data will change to some extent, which will affect the accuracy of the data in the study. However, it will not affect the research conclusions related to the developmental characteristics of China’s legal system, the range of public health and quality of life, and the expansion of the government’s administrative power during the pandemic. Second, influenced by the research focus, when selecting research samples related to China’s rule of law system for research, the focus was on selecting samples that were closely related to public health and quality of life. It may be difficult to show the overall picture of China’s rule of law system during the COVID-19 pandemic.

## 6. Conclusions

As seen during the COVID-19 pandemic, safeguarding people’s life, health, and safety has always been an important concept in China’s legal system. The legal system for pandemic prevention and control provides a layered legal guarantee of the quality of public health and life during a pandemic with a legislative system, administrative law enforcement mechanism, and judicial trial mechanism. The legislative system clarifies the public’s right to a healthy life during a pandemic and provides a basis for government departments to carry out pandemic prevention and control. The administrative law enforcement mechanism is used to prevent illegal administrative acts from adversely affecting the social public order during a pandemic, and to stop and punish disruptive acts in a timely manner. The judicial trial mechanism provides a legal guarantee of the public’s healthy quality of life in three ways: punishing criminal acts, protecting legitimate civil rights and interests, and supervising the administrative bodies to make sure they perform their duties according to the law. After the outbreak of SARS-CoV-2, the quality of public health and life was adversely affected in many aspects. China’s rule of law system engaged in many legal activities through the legislative system, administrative law enforcement mechanism, and judicial trial mechanism, maximizing the value of different legal means to protect people’s health and maintain social order. There is a close relationship between the social order and the legal system, so it also reflects the important role of the rule of law system in ensuring the public’s healthy quality of life to a large extent.

The Chinese government maximized the value of the rule of law in maintaining public order and safeguarding the legitimate rights of the public under emergency conditions. By improving the legal system, the administrative supervision capacity, and the judicial review mechanism under emergency conditions and other emergency management and control methods, the government established a rule of law system to ensure people’s health and quality of life. This legal protection was not limited to pandemic prevention and control, but also involved the economic order, living environment, and personal development needs that affect the satisfaction and security of public life, reflecting a consistency between the rule of law and the quality of public health and life. At the same time, government departments often take temporary emergency measures based on the consideration of pandemic prevention and control [33]. If these measures are not effectively regulated, they will adversely impact the quality of public health and life [34], thus creating new contradictions between the government’s emergency pandemic management activities and the quality of public health and life. At this time, China’s legal system has been entrusted with the responsibility of coordinating such contradictory demands.

On the one hand, China’s rule of law system, by expanding the scope of the government’s administrative management activities under emergency conditions, maximized the value of such activities for pandemic emergency management, thereby reducing the impact of the pandemic on public health and quality of life. On the other hand, the quality of health and life is also reflected in interaction and cooperation between the public and the government, based on the common goal of pandemic prevention and control [35]. In addition, since the quality of public health and life has had a close relationship with the government’s administrative behavior during the COVID-19 pandemic, to avoid the adverse impact of the government’s illegal actions in the future, strengthening the interactions between social subjects and administrative subjects and ensuring the legitimacy of the administrative activities of government departments under emergency conditions will also be main concerns in further development of China’s legal system.

## Figures and Tables

**Table 1:** Number of laws in China’s legal system related to pandemic prevention and control.

Legal System	Issuing Authority	Before SARS-CoV-2 Outbreak (%)	After SARS-CoV-2 Outbreak (%)	Total
Laws	National People’s Congress and its Standing Committee	13 (59.1)	9 (40.9)	22
Administrative regulations	State Council	18 (81.8)	4 (18.2)	22
Judicial interpretation	Supreme People’s Court and Supreme People’s Procuratorate	5 (83.3)	1 (16.7)	6
Departmental rules	Department of the State Council	112 (88.2)	15 (11.8)	127
Local regulations	Local People’s Congress and its Standing Committee	322 (49.8)	325 (50.2)	647
Government regulations	Local People’s Government	227 (73.2)	83 (26.8)	310

**Table 2:** Number of documents in normative document system related to pandemic prevention and control in China.

Issuing Authority	Before SARS-CoV-2 Outbreak (%)	After SARS-CoV-2 Outbreak (%)	Total
State Council	152 (54.5)	127 (45.5)	279
Department of the State Council	1226 (59.5)	835 (40.5)	2061
Local People’s Congress and its Standing Committee, and People’s Government	9472 (38.7)	14,998 (61.3)	24,470

**Table 3:** Number of documents in working document system related to pandemic prevention and control in China.

Issuing Authority	Before SARS-CoV-2 Outbreak (%)	After SARS-CoV-2 Outbreak (%)	Total
National People’s Congress and its Standing Committee	21 (30.9)	47 (69.1)	68
Supreme People’s Court	0 (0)	42 (100)	42
Supreme People’s Procuratorate	0 (0)	7 (100)	7
Department of the State Council	1444 (31.6)	3119 (68.4)	4563
Local People’s Congress and its Standing Committee, and People’s Government	20,128 (32.8)	41,292 (67.2)	61,420

**Table 4:** Number of laws and relevant documents on quality of life involving public health.

	Legal System	Normative Document	Working Document	Total
Hygiene	236	7785	12,559	20,580
Pandemic prevention and control	90	10,555	8436	19,081
Business environment optimization	38	1067	4164	5269
Education	11	974	3508	4493
Agriculture	74	1140	2512	3726
Labor union	6	831	2025	2862
Construction industry	28	645	2093	2766
Civil administration	12	757	1542	2311
Public security	115	471	1540	2126
Finance	4	601	1273	1878
Sports	4	176	1476	1656
Legal work	32	503	1070	1605
Transportation	19	493	1004	1516
Science and technology	2	182	1258	1442
Animal husbandry	30	546	836	1412
Forestry	93	396	862	1351
Commercial and trading partners	15	360	924	1314
Enterprise	1	553	652	1206
Tourism	5	258	907	1170
Environmental protection	43	250	779	1115
Business administration	16	454	614	1084
Tax revenue	1	419	560	980
Price	7	496	356	859
Real estate	9	273	540	822
Quality management and supervision	1	190	626	817
Culture	3	124	613	740
Commodity inspection, animal and plant quarantine	51	421	240	712
Food services	2	229	343	574
Postal communication	8	94	342	444
Fisheries	18	75	185	278

**Table 5:** Number of administrative law enforcement activities by government departments during COVID-19 pandemic.

	Before SARS-CoV-2 Outbreak	After SARS-CoV-2 Outbreak	Total
Pandemic prevention and control	102	11,926	12,028
Public security	33	9143	9176
Market supervision	76	6846	6922
Food, medicine, and medical treatment	372	2913	3285
Finance and tax	0	1527	1527
Internet	10	565	575
Transportation	0	558	558
Agriculture, forestry, fisheries, and animal husbandry	91	271	362
Land and urban construction	3	351	354
Environmental protection	1	236	237
Culture and tourism	0	95	95
Safety supervision	1	83	84
Public culture and media	0	45	45
Intellectual property rights	2	38	40
Civil affairs and labor social security	0	15	15
Education	0	10	10
Sports	0	6	6
Water conservancy	1	4	5

**Table 6:** Number of criminal cases involving the pandemic situation.

	Before SARS-CoV-2 Outbreak	After SARS-CoV-2 Outbreak	Total
Property invasion	52	13,001	13,053
Hindering social order	190	9257	9447
Endangering public security	11	7423	7434
Disrupting order of market economy	241	4161	4402
Violating citizens’ personal and democratic rights	10	4013	4023
Corruption and bribery	131	571	702
Malfeasance	182	145	327

**Table 7:** Number of civil cases involving pandemic situation.

	Before SARS-CoV-2 Outbreak	After SARS-CoV-2 Outbreak	Total
Contract dispute	4523	319,940	324,463
Labor dispute	209	43,920	44,129
Intellectual property and competition	15	22,112	22,127
Infringement dispute	327	16,113	16,440
Right in rem	135	7435	7570
Special procedure cases	10	5228	5238
Disputes over personality rights	163	4307	4470
Marriage and family	25	4180	4205
Company dispute	6	3374	3380
Insurance dispute	35	2271	2306
Unjust enrichment	11	1549	1560
Inheritance	2	1172	1174
Maritime affairs	7	284	391

**Table 8:** Number of administrative cases involving pandemic situation.

	Before SARS-CoV-2 Outbreak	After SARS-CoV-2 Outbreak	Total
Labor and social security	20	963	983
House demolition	24	421	445
Township government	58	299	357
Administrative supervision	2	318	320
Public security	4	308	312
Urban construction	1	215	216
Quality supervision	3	200	203
Transportation	1	157	158
City planning	0	118	118
Housing registration	0	96	96
Hygiene	20	75	95
Civil administration	0	84	84
Environmental protection	4	74	78
Judicial administration	0	71	71
Agriculture	16	45	61
Education	19	38	57
Food and medicine	3	18	21
Culture	0	6	6
Sports	0	3	3

## Data Availability

Not applicable.
